# Luxation of Femur on Dorsum Ilii—Reduction by Manipulation

**Published:** 1879-11

**Authors:** Wm. C. Phelps


### Clinical Reports.

---

### LUXATION OF FEMUR ON DORSUM ILII—REDUCTION BY MANIPULATION.

REPORTED BY WM. C. PHELPS, M. D.

F. W. H., Jr., a vessel captain, aged 41, a robust and powerful man, was brought to the Buffalo General Hospital, on the night of July 2d last, at about twelve o'clock, having a dislocation of the right femur on the dorsum of the ilium. The accident occurred about four hours previous to his admission, in the following manner: He started to go down from the third floor of a building, and just as he reached the landing of the stairs, his foot caught in a piece of matting, which threw him down about five of the steps, his whole weight coming on the right foot, which drove the head of the femur upward on the dorsum.

It was a typical case of the injury, all the usual diagnostic signs being present—shortening, inversion of the foot, flexion of the thigh on the body, mal-position of the trochanter major, and head of the bone, which could be plainly felt on the dorsum, loss of voluntary motion, &c. Attempts at reduction had been made by two medical men by manipulation and extension before his admission, but without success. The patient was etherized by the resident physician, Dr. Sheldon, while lying on an operating table, and I attempted reduction by the usual method of manipulation, but failed. I then removed him to the floor, and proceeded to use *upward traction*, in addition to manipulation, as directed by Bigelow. I removed my left shoe, and after having flexed the leg on the thigh, and the thigh to a right angle with the body, placed my foot on the anterior superior spinous process of the injured side, passed my left arm under the flexed leg, and with my right hand firmly grasped the ankle. I now made strong but steady traction directly upward, at the same time gently rotating the femur, by moving the ankle from side to side, and in a few seconds the thigh began to move upwards, and after having traveled, it seemed to me, at least eight or ten inches, it suddenly slipped into the acetabulum, with a plainly audible sound. The whole operation did not extend over one minute of time. I then placed the injured limb beside the sound one, and found it entirely normal in length and position. He was placed in a bed, and in three weeks was about on crutches.

August 8th. He was around the hospital grounds without any support.

During his convalescence he complained considerably of lameness of his back, a slight complication of his injury.

I believe this case illustrates the value of the manœuvres, recommended by Bigelow, of upward traction in certain cases, and it may be of service to the profession to call their attention to it in this manner, in fact, the only thing in the case worthy of note.

